# Utility of Select Gene Mutation Detection in Tumors by the Idylla Rapid Multiplex PCR Platform in Comparison to Next-Generation Sequencing

**DOI:** 10.3390/genes13050799

**Published:** 2022-04-29

**Authors:** Dingani Nkosi, Vektra L. Casler, Chauncey R. Syposs, Zoltán N. Oltvai

**Affiliations:** Department of Pathology and Laboratory Medicine, University of Rochester School of Medicine & Dentistry, Rochester, NY 14642, USA; dingani_nkosi@urmc.rochester.edu (D.N.); vektra_casler@urmc.rochester.edu (V.L.C.); chauncey_syposs@urmc.rochester.edu (C.R.S.)

**Keywords:** next generation sequencing, Idylla platform, molecular diagnostics

## Abstract

Testing of tumors by next generation sequencing (NGS) is impacted by relatively long turnaround times and a need for highly trained personnel. Recently, Idylla oncology assays were introduced to test for *BRAF*, *EGFR*, *KRAS*, and *NRAS* common hotspot mutations that do not require specialized trained personnel. Moreover, the interpretation of results is fully automated, with rapid turnaround time. Though Idylla testing and NGS have been shown to have high concordance in identifying *EGFR*, *BRAF*, *KRAS*, and *NRAS* hotspot mutations, there is limited experience on optimal ways the Idylla system can be used in routine practice. We retrospectively evaluated all cases with *EGFR*, *BRAF*, *KRAS*, or *NRAS* mutations identified in clinical specimens sequenced on two different NGS panels at the University of Rochester Medical Center (URMC) molecular diagnostics laboratory between July 2020 and July 2021 and assessed if these mutations would be detected by the Idylla cartridges if used. We found that the Idylla system could accurately identify Tier 1 or 2 actionable genomic alterations in select associated disease pathologies if used. Yet, in a minority of cases, we would have been unable to detect NGS-identified pathogenic mutations due to their absence on the Idylla panels. We derived algorithmic practice guidelines for the use of the Idylla cartridges. Overall, Idylla molecular testing could be implemented either as a first-line standalone diagnostic tool in select indications or for orthogonal confirmation of uncertain results.

## 1. Introduction

Development of personalized therapies for cancer patients in the last two decades has had a substantial impact on the treatment strategies of different tumor types [1]. Testing to detect genomic alterations in tumors is standard of care in establishing diagnosis, for calculating prognosis, and for selecting optimal therapies. Some of the commonly identified mutations associated with targeted therapies include, e.g., mutations in *BRAF* in cutaneous melanoma; *KRAS*, *BRAF*, and *NRAS* in colorectal cancer; and *EGFR* mutations and *ALK* and *ROS1* gene rearrangements in lung adenocarcinomas [2,3,4].

The gold standard for detecting different genomic alterations in tumors is using next generation sequencing (NGS)-based assays [5]. Institutions implement different NGS workflow plans depending on the NGS test available to them (i.e., gene panel list) and sample volume. However, relatively low sample volume for NGS testing and the specifics of standard NGS testing platforms typically requires sample batching, with suboptimal turnaround times of about 12–15 days. These are also high-complexity, labor-intensive tests that require highly trained specialized personnel; thus, they are not immediately available at smaller institutions and hospitals.

Recently, Idylla^TM^ oncology assays (Biocartis, Mechelen, Belgium) were launched to complement NGS testing. The Idylla system is an allele-specific qPCR-based assay platform in which all wet-bench steps are automated and confined to a single use cartridge. The assay starts after the insertion of sample-loaded gene(s) specific cartridges into the unit that are connected to a user interface console that displays result summaries. qPCR curve results generated from the assay are visualized through a secure web-based interface. The Idylla BRAF, EGFR, KRAS, and NRAS/BRAF mutation tests (cartridges) on the Idylla platform allow the detection of *EGFR*, *BRAF*, *NRAS* and *KRAS* hotspot mutations with rapid turnaround times (<3 h). Unlike NGS-based assays, the Idylla system can directly use formalin-fixed paraffin-embedded (FFPE) tissue sections without the need for DNA preparation and extraction, with interpretation of the results being fully automated. Several studies have demonstrated the validity and accuracy of the Idylla system in comparison to NGS in detecting *EGFR*, *BRAF*, *KRAS*, and *NRAS* hotspot mutations [6,7,8,9,10,11,12]. Yet, despite the high concordance of the Idylla and the NGS results, little has been done to formally assess how to best use the Idylla system clinically as a first-line diagnostic tool.

The overall objective of this study has been to start developing a formalized workflow on the optimal use of the Idylla system as a first-line diagnostic tool for the detection of different actionable genomic mutations in routine clinical practice. To this end, we retrospectively identified cases with *EGFR*, *BRAF*, *KRAS*, or *NRAS* mutations detected in specimens that were sequenced on two different NGS panels at the University of Rochester Medical Center (URMC) molecular diagnostics laboratory and assessed if the mutations would be detected on the Idylla system cartridges. The *EGFR*, *BRAF*, *KRAS*, or *NRAS* NGS detected mutations were compared to mutations that could be identified by the Idylla cartridges if used. We classified gene mutations identified by the NGS panels according to their tier grouping and evaluated to what extent the Idylla platform would have detected these mutations if utilized. Furthermore, we selected NGS-diagnosed *BRAF* or *NRAS* mutation positive cutaneous melanomas, *BRAF* mutation positive hairy cell leukemia, *KRAS* or *EGFR* mutation positive lung adenocarcinomas, and *BRAF*, *KRAS* or *NRAS* mutation positive colorectal cancer cases to assess what proportion of the identified mutations would have been recognized on the Idylla platform. Lastly, we also uncovered a proportion of NGS-evaluated cases with mutations that would have been detected on the Idylla system but had additional, Idylla non-identifiable mutations.

## 2. Materials and Methods

### 2.1. Clinical Specimens for Amplicon Based NGS Testing

We retrieved NGS results from all specimens referred for routine testing at the URMC Molecular diagnostics laboratory between July 2020 and July 2021. These samples were tested using laboratory-developed NGS-based assays using either the ThermoFisher’s Oncomine Focus Assay panel (Table S1) or the Illumina TruSight^TM^ Myeloid sequencing panel (Table S1). From the database, we grouped the results to analyze the identified gene mutations in *BRAF*, *KRAS*, *EGFR,* and *NRAS* genes. The nucleotide changes identified for the four genes were compared to detectable *BRAF*, *KRAS*, *EGFR*, and *NRAS* variants on the Idylla cartridges.

### 2.2. cBioPortal for Cancer Genomics

Point mutation data was collected from the “cBioPortal for Cancer Genomics” website (https://www.cbioportal.org/datasets, accessed on or about 15 December 2021) for all studies with greater than 500 recorded point mutations. These data included the following studies: [13,14,15,16,17,18,19,20] Zehir et al. [13], Zhang et al. [14], Bolton et al. [15], Pereira et al. [16], Stopsack et al. [17], Razavi et al. [18], Samstein et al. [19], Nguyen et al. [20], Myelodysplastic (MSKCC, 2020), Cancer Cell Line Encyclopedia (Broad, 2019), MSK-IMPACT and MSK-ACCESS Mixed Cohort (MSK, 2021), Li et al. [21], Campbell et al. [22], Yaeger et al. [23], Pediatric Neuroblastoma (TARGET, 2018), Breast Invasive Carcinoma (TCGA, PanCancer Atlas), Ho et al. [24], Barretina et al. [25], Armenia et al. [26], Jonsson et al. [27], Reddy et al. [28], Jordan et al. [29], Grobner et al. [30], Ciriello et al. [31], Ceccarelli et al. [24,25,26,27,28,29,30,31,32], Mature B-cell malignancies (MD Anderson Cancer Center), Melanoma (MSKCC, 2018), Tyner et al. [33], Giannakis et al. [34], Lung Adenocarcinoma (MSKCC, 2020), Lung Adenocarcinoma (TCGA, PanCancer Atlas), Landau et al. [35], Colorectal Adenocarcinoma (TCGA, PanCancer Atlas), Ovarian Serous Cystadenocarcinoma (TCGA, PanCancer Atlas), Kim et al. [36], Uterine Corpus Endometrial Carcinoma (TCGA, PanCancer Atlas), Head and Neck Squamous Cell Carcinoma (TCGA, PanCancer Atlas), Brain Lower Grade Glioma (TCGA, PanCancer Atlas), and Head and Neck Squamous Cell Carcinoma (TCGA, Firehose Legacy), Koboldt et al. [37], Puente et al. [38], Abida et al. [39]. Records from these studies were aggregated and filtered, with only data columns for ‘study name’, ‘specimen ID’, ‘gene target’, ‘codon mutation name’ (example: c.901C>G), ‘long protein mutation name’ (example: p.Ala379Val), and ‘short protein mutation name’ (example: p.A379V) retained. Duplicate entries were filtered out, leaving approximately 3.7 million point-mutation records. The data was further filtered to include only four genes of interest: *BRAF*, *EGFR*, *KRAS*, and *NRAS*. This created a list of 11,420 unique ‘study-sample’ concatenation records.

A list of Idylla-identifiable point mutation targets was created based on package insert materials (comprising a list of 196 unique point mutations and indels spread across the 4 target genes). We designed a Python programming language script to compare each of the Idylla-identifiable mutations against each entry in the ‘four-target-gene-only’ data aggregate from the selected cBioPortal studies. We included successful matches in a list of ‘study-sample’ string-concatenations. Lastly, we filtered these results for unique values to avoid over-counting specimens which may have had multiple Idylla-identifiable mutations. We then compared the portion of Idylla-identifiable point mutations to the total number of ‘four-target-genes-only’ mutations found in the prepared cBioPortal data.

### 2.3. Statistical Analysis

Data analysis and figures were created by using Microsoft Excel, GraphPad 8.3, and CorelDraw 2019 software programs.

## 3. Results

### 3.1. Idylla Cartridges Can Detect Most NGS Test Identified Tier 1 and Tier 2 Hotspot Mutations in EGFR, BRAF, KRAS, and NRAS

In the Idylla system, all assay cartridges are ready-to-use and contain the necessary reagents to perform sample preparation and real-time PCR amplification and detection, starting from insertion of FFPE tissue sections into the cartridges. The detectable variants for each target gene by the Idylla BRAF, KRAS, EGFR, and NRAS/BRAF cartridges are listed in Table S2 or on technical data sheets from https://www.biocartis.com/en-US/meet-idylla/idylla-oncology-assays (accessed on 29 December 2021).

We collated a total of 159 tumor samples bearing *BRAF* mutations, 133 with *NRAS* mutations, 303 with *KRAS* mutations, and 96 with *EGFR* mutations from the URMC NGS database from June 2020 to July 2021. We compared the NGS assay-detected nucleotide (protein) changes on these genes to the Idylla-detectable variants for these four genes (Table S2).

**Figure 1:**
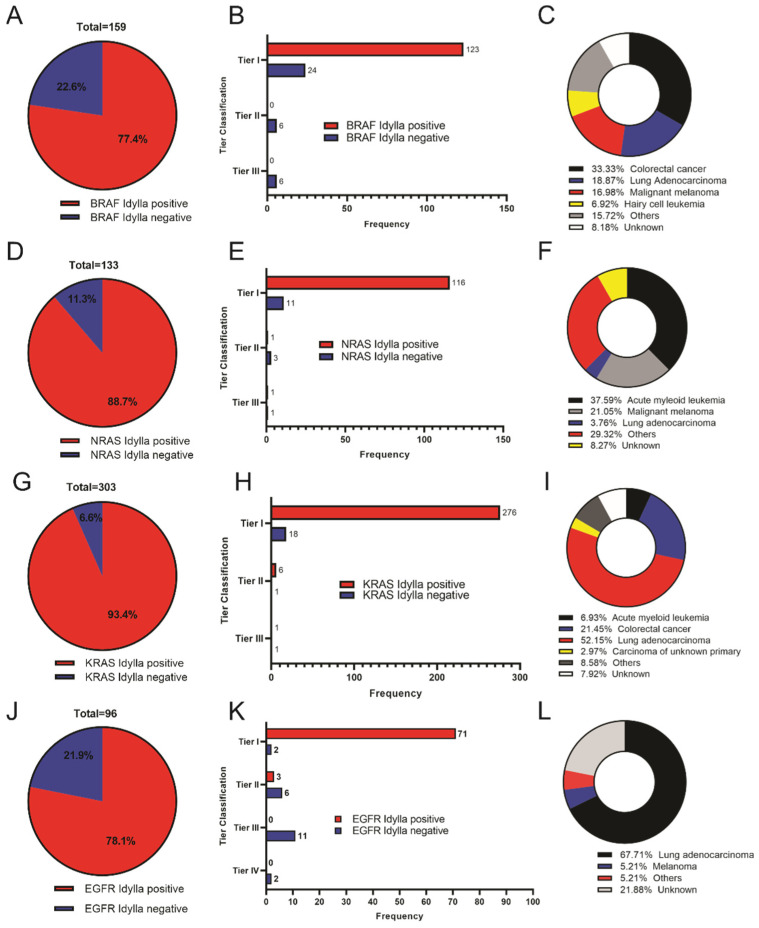
Proportion of gene mutations identified on NGS that would be positive on Idylla molecular testing. (**A**–**C**) Percentage of *BRAF* mutations identified on NGS panel that would be detected on Idylla system and associated Tier classification and primary site diagnosis. (**D**–**F**) Percentage of *NRAS* mutations identified on NGS panel that would be detected on Idylla system and associated Tier classification and primary site diagnosis. (**G**–**I**) Percentage of *KRAS* mutations identified on NGS panel that would be detected on Idylla system and associated Tier classification and primary site diagnosis. (**J**–**L**) Percentage of *EGFR* mutations identified on NGS panel that would be detected on Idylla system and associated Tier classification and primary site diagnosis.

For *BRAF*, 77.4% of the variants identified on the NGS panels could potentially be identified by the Idylla system, while the remaining 22.6% could not (Figure 1A). We categorized 93% (147 of 159) of these *BRAF* mutations as Tier 1 mutations, of which 84% (123 of 147) could likewise be identified by the Idylla system (Figure 1B) if used. The majority of the samples with *BRAF* mutations were from colorectal cancers (approximately 33%) followed by lung adenocarcinoma (19%) and malignant melanoma (17%) (Figure 1C).

For the identified *NRAS* mutations, 89% (118 of 133) of the variants identified by NGS testing could be identified on the Idylla system as well (Figure 1D). 91% of the Tier 1 *NRAS* mutations (116 of 127) could be detected by the Idylla NRAS cartridge if used (Figure 1E). Most specimens with *NRAS* mutations were from acute myeloid leukemia patients (approximately 38%), followed by malignant melanoma (21%) and lung adenocarcinoma (4%) (Figure 1F).

*KRAS* mutation analysis revealed that 93% (283 of 303) of the NGS-detected mutations could be identified by the Idylla system (Figure 1G). We classified 294 of the *KRAS* mutations to be Tier 1, of which 276 (94%) would be detected on the Idylla system if utilized (Figure 1H). The majority of the samples were from lung adenocarcinoma (~52%), followed by colorectal cancer (21%) (Figure 1I).

*EGFR* mutation evaluation showed that 78% (76 of 96) of the NGS-identified *EGFR* mutations could likewise be detected by the Idylla system if run (Figure 1J). Furthermore, the Idylla system, if utilized, is predicted to have identified 97% (71 of 73) of the NGS-detected Tier 1 *EGFR* mutations (as shown by the upper bars of Figure 1K). Most of the *EGFR* mutations were from lung adenocarcinoma, at about 68%, and malignant melanoma, approximately 5% (Figure 1L).

Taken together, our results show that the four DNA extraction-based Idylla cartridges could identify most of the Tier 1 mutations detected by routine NGS testing if used.

### 3.2. Idylla Cartridges Identify Most High Tier Mutations in Samples from cBioportal for Cancer Database

Additionally, we compared the single nucleotide variant (SNV) and small indel profiles identifiable by NGS testing at the URMC Molecular Diagnostics laboratory for *EGFR*, *BRAF*, *KRAS*, and *NRAS* genes to their comprehensive variant profiles, using data for these genes compiled from the cBioPortal for Cancer Genomics website (https://www.cbioportal.org/datasets, accessed on 29 December 2021). The data comprised a total of 11,420 unique samples with either *EGFR*, *BRAF*, *KRAS,* or *NRAS* gene mutation profiles, spread across 44 studies (see Methods). This data set contained 2483 samples with *BRAF* mutations and 2960 with *EGFR* mutations, of which 1346 (54%) *BRAF* mutations and 1122 (38%) *EGFR* mutations could be identified by the Idylla system if used (Table 1). Furthermore, a total of 4667 samples with *KRAS* and 1636 with *NRAS* mutations from the cBioPortal data were compared to the Idylla system-detectable targets. The Idylla platform could have identified 4232 (91%) *KRAS* and 1423 (87%) *NRAS* mutations as positive if utilized (Table 1).

### 3.3. Assessment of the Idylla System with Mutations Linked to Specific Disease Pathologies

The utility of the Idylla system may vary from one tumor type to another. Thus, to determine the type and frequency of gene mutations that would be missed by the Idylla system, we next grouped gene alterations with their associated disease pathologies. We hypothesized that associating the NGS-identified gene mutations to their specific associated disease pathologies (e.g., lung adenocarcinoma with *EGFR* mutations or cutaneous melanoma with *NRAS* or *BRAF* mutations) would demonstrate higher concordance with hotspot mutations recognized by the Idylla system and may also uncover additional clinically significant NGS-identified gene mutations not detected by Idylla. The comprehensive results are detailed in Tables S3–S10.

We identified 27 cutaneous melanoma cases with *BRAF* mutations, of which 21 (78%) could also be detected by the Idylla BRAF and BRAF/NRAS cartridges if run (Table 2). Eight (33%) of the cutaneous melanoma samples had additional NGS-identified mutations (Table 2), of which four possessed *BRAF* variants that cannot be identified by the Idylla system, two of which were Tier 1 mutations. In the remaining three cases, one had a Tier 1 *MAP2K1* mutation while the rest of the variants were mostly classified as Tier 3 (Table 2 and Table S3).

*NRAS* mutations were identified in 28 cutaneous melanoma cases, of which 26 (93%) could be also detected if run on the Idylla system (Table 2). Twelve (43%) *NRAS* mutation bearing cutaneous melanoma cases had additional mutations, with two harboring Tier 1 *NRAS* mutations that cannot be identified by the Idylla system (Table 2). Five of the other extra mutations were classified as Tier 1, while the remaining four variants were Tier 3 mutations (Table 2 and Table S4).

BRAF V600E mutations are present in almost all cases of hairy cell leukemia (HCL) at diagnosis [40]. Indeed, ten of the eleven HCL cases had *BRAF* V600E mutations that would be detected on the Idylla system if utilized (Table 2). Three (30%) HCL cases had additional genomic alterations, two of which (a *TET2* and *SF3B1* mutation) were classified as Tier 1 (Table 2 and Table S5).

We identified 53 patients with *BRAF* mutation bearing colorectal cancer (CRC), of which 51 (96%) had NGS identified *BRAF* mutations that would be detected on the Idylla system if performed (Table 2). Seventeen (32%) cases were observed to have additional mutations. Eight of these cases had one or more Tier 1 mutation(s) while the rest of the cases mostly had Tier 3 mutations (Table 2 and Table S6).

*KRAS* mutations were also identified in 65 CRC cases, of which 62 (95%) could be identified on the Idylla system if run (Table 2). We identified 24 (37%) cases with additional mutations (Table 2). Seventeen of these cases had one or more Tier 1 mutation(s), with *PIK3CA* alterations being the mostly frequently observed (Table 2 and Table S7).

*NRAS* mutations were also identified in three cases with CRC and two (66%) of the identified genomic alterations could be identified on the Idylla system (Table 2). We identified two cases with additional Tier 1 *BRAF* and *PIK3CA* mutations (Table 2 and Table S8).

Mutated *KRAS* and *EGFR* are common oncogenic drivers of lung adenocarcinoma with predictive value for targeted therapies [41,42]. Most *EGFR* mutations occur within its kinase domain, encoded by exons 18–21 [43]. We observed 65 cases of lung adenocarcinoma with *EGFR* mutations, of which 56 (86%) could be identified using the Idylla EGFR cartridge (Table 2) if used. Twenty-one (32%) samples harbored additional mutations, of which nine cases harbored a Tier 1 mutation (Table 2 and Table S9).

*KRAS* mutations were identified in 158 lung adenocarcinoma samples, of which 147 (93%) could be identified using the Idylla KRAS cartridge (Table 2). Twenty-five (13%) of the cases had extra mutations identified (Table 2), of which twelve possessed a Tier 1 mutation (Table 2 and Table S10).

Altogether, these data show that NGS identified *EGFR*, *BRAF*, *KRAS*, and *NRAS* mutations with select associated disease pathologies displayed high, but imperfect, concordance with mutations that would be detected by the Idylla cartridges if used, as a substantial minority of Idylla positive cases harbored additional NGS-identified Tier 1 or 2 gene mutations.

## 4. Discussion

NGS assays represent the gold standard for identifying genomic mutations in tumors [5]. However, introducing novel, closed-system technologies, such as the fully automated Idylla system, is of paramount importance because they can shorten the turnaround time and do not require specially trained laboratory personnel for their use.

Idylla cartridges are intended to identify common, clinically relevant Tier 1 and 2 mutations, hence they do not detect rare or complex variants. Our analyses and the results of other studies verified the Idylla system’s capacity for highly accurate detection of Tier 1 or 2 *BRAF*, *NRAS*, *KRAS*, and *EGFR* mutations within different malignancies [9,12,44,45,46]. Similarly, Van Haele et al. showed that *BRAF*, *EGFR*, and *KRAS* mutation testing on the Idylla system had a very high overall concordance with the TruSight Tumor26 NGS panel on actionable genomic mutations [10]. The lower detection levels for *BRAF* and *EGFR* mutations on the Idylla system with the cBioPortal for Cancer Genomics dataset can be attributed to the fact that most of the identified variants are rare and/or non-pathogenic. Thus, these variants would be outside the Idylla mutation profile range. Our data further demonstrated that NGS-detected *BRAF*, *NRAS*, *KRAS*, and *EGFR* mutations with select associated disease pathologies had high similarity with mutations that could be identified by Idylla cartridges. These data suggest that the Idylla system can be implemented as a standalone diagnostic tool or can be used as an orthogonal tool to confirm results from other molecular diagnostic assays.

**Figure 2:**
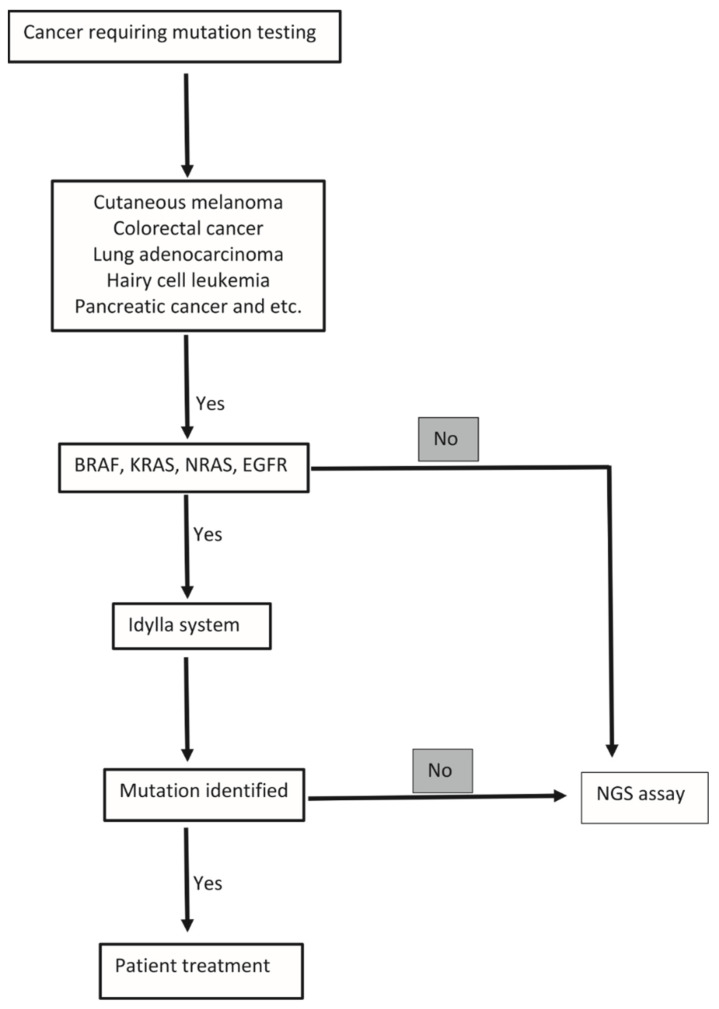
Generic algorithm for the clinical use of the Idylla platform.

**Figure 3:**
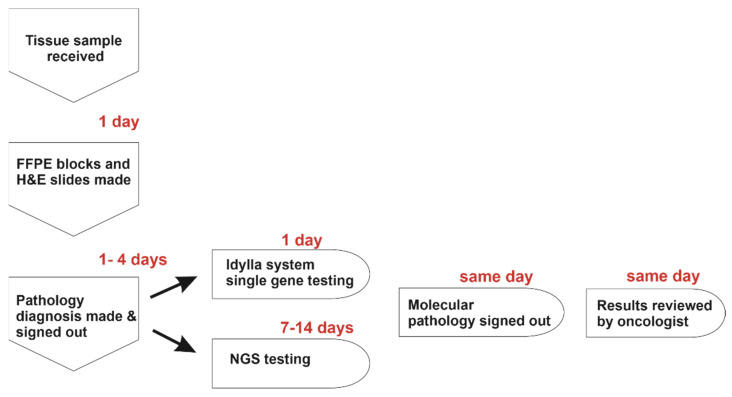
Algorithm for processing of tumor samples for molecular testing.

From this, we derive a proposed algorithm on how the Idylla can be best utilized as a standalone diagnostic tool when testing for these select gene mutations (Figure 2). We also outline an algorithm of processing tumor samples for routine molecular testing (Figure 3). Pathologists in smaller and/or rural hospitals without an on-site molecular laboratory can easily incorporate and perform these molecular tests on FFPE tissue sections, obviating the need for DNA preparation and extraction while also offering the fully-automated interpretation of these results [47]. If no mutation is identified, the sample can be reflexed to send-out NGS testing. Alternatively, in large academic medical centers, the Idylla system can be used for orthogonal confirmation of ambiguous results from other (NGS) testing methods or for on-demand single gene testing when rapid identification of *BRAF*, *NRAS*, *KRAS*, and *EGFR* mutations for diagnosis and/or therapeutic decisions is required (Figure 3). Indeed, studies have shown that the Idylla molecular testing report can be reported within a day while initiating, e.g., EGFR-targeted treatments in NSCLC [48].

Though the Idylla system is a valuable tool for identifying select pathogenic mutations, it also has some considerable limitations. Tumor samples bearing complex mutations pose a challenge, as the Idylla system only identifies a limited number of pathogenic mutations while other pathogenic mutations outside the hotspots of the listed genes would not be identified. We have also shown that a substantial minority of samples have more than one Tier 1 and/or 2 mutations where the Idylla system would not identify all pathogenic mutations present in the sample. Samples with such mutation patterns present a conundrum: whether all samples initially run on the Idylla system should be reflexed for NGS testing, despite detecting an actionable mutation. For example, the *IDH1* mutations we detected by NGS in Idylla-detectable *BRAF* or *KRAS* mutation positive CRC specimens (Tables S6 and S7) are associated with mucinous or signet ring cell adenocarcinoma, thus providing molecular support for the correct diagnosis that would not be possible from the Idylla results [49]. The Idylla system has also been shown to be ineffective in identifying some drug-resistant mutations when present at low variant allele levels within the tumor [48]. An additional shortcoming of the Idylla system is that it can test only one sample at a time per instrument, limiting throughput [9]. It is also worth noting that the choice to utilize the Idylla molecular testing can be influenced by sample size. Scant tissue samples may necessitate conventional NGS protocols.

Taken together, our analyses have shown that the Idylla platform is able to identify the majority of common pathogenic mutations in the *BRAF*, *NRAS*, *KRAS*, and *EGFR* genes, has a very fast turnaround time, and does not require specialized personnel training—all of which makes it an ideal diagnostic system in small hospital/remote area settings. In contrast, for larger institutions with relatively fast NGS capabilities, the Idylla system can be best utilized to support select morphologic diagnoses (e.g., identifying BRAF V600E mutation to support diagnosis of hairy cell leukemia) or for orthogonal confirmation of uncertain or very low allele frequency mutation results.

## Figures and Tables

**Table 1:** cBioPortal for Cancer Genomics and associated gene mutations.

Gene	Idylla Positive	Idylla Negative
*BRAF*	1346 (54%)	1137 (46%)
*EGFR*	1122 (38%)	1838 (62%)
*KRAS*	4232 (91%)	435 (9%)
*NRAS*	1423 (87%)	213 (13%)

**Table 2:** Gene mutations and associated specific disease.

Gene Mutation and Diagnosis	Case Numbers	Idylla Positive	Idylla Negative	Cases with Extra Mutations	Cases with Extra and Tier 1 Mutations Not Detected by Idylla
*BRAF* melanoma	27	21 (78%)	6 (22%)	8 (30%)	2 (8%)
*NRAS* melanoma	28	26 (93%)	2 (7%)	12 (43%)	5 (18%)
*BRAF* HCL	11	10 (91%)	1(9%)	3 (27%)	3 (27%)
*BRAF* CRC	53	51 (96%)	2 (4%)	17 (32%)	9 (17%)
*KRAS* CRC	65	62 (95%)	3 (5%)	24 (37%)	17 (26%)
*NRAS* CRC	3	2 (66%)	1 (33%)	2	2 (66%)
*EGFR* lung adenocarcinoma	65	56 (86%)	9 (7%)	21 (32%)	7 (11%)
*KRAS* lung adenocarcinoma	158	147 (93%)	11 (7%)	25 (16%)	12 (8%)

## Data Availability

The consent documentation signed by the patients do not expressly allow submission of full sequencing data (FASTQ, BAM/BAI, VCF) to external data repositories.

## Supplementary Materials

The following supporting information can be downloaded at: https://www.mdpi.com/article/10.3390/genes13050799/s1, Tables S1–S10. Table S1: (A) Oncomine Focus Assay (OFA) hotspot gene list; (B) TruSeq myeloid panel gene list; Table S2: (A) Idylla BRAF cartridge detectable mutations; (B) Idylla EGFR cartridge detectable mutations; (C) Idylla KRAS cartridge detectable mutations; (D) Idylla NRAS-BRAF cartridge detectable mutations; Table S3: Cutaneous melanoma specimens with Idylla-identifiable *BRAF* mutations and/or with additional genetic alterations; Table S4: Cutaneous melanoma specimens with Idylla-identifiable *NRAS* mutations and/or with additional genetic alterations; Table S5: Hairy cell leukemia specimens with Idylla-identifiable *BRAF* mutations and/or with additional genetic alterations; Table S6: Colorectal cancer specimens with Idylla-identifiable *BRAF* mutations and/or with additional genetic alterations; Table S7: Colorectal cancer specimens with Idylla-identifiable *KRAS* mutations and/or with additional genetic alterations; Table S8: Colorectal cancer specimens with Idylla-identifiable *NRAS* mutations and/or with additional genetic alterations; Table S9: Lung adenocarcinoma specimens with Idylla-identifiable *EGFR* mutations and/or with additional genetic alterations; Table S10: Lung adenocarcinoma specimens with Idylla-identifiable *KRAS* mutations and/or with additional genetic alterations.
