# Supplementary material for: Utility of Select Gene Mutation Detection in Tumors by the Idylla Rapid Multiplex PCR Platform in Comparison to Next-Generation Sequencing
Source: Genes (Basel). 2022 Apr 29;13(5):799. doi: 10.3390/genes13050799 (PMC9141835; doi:10.3390/genes13050799)
Supplement: Supplementary file 1 [file genes-13-00799-s001.zip › genes-1613678-supplementary.pdf]

Supplemental Tables

Utility of select gene mutation detection in tumors by the Idylla rapid multiplex PCR platform in comparison to next-generation sequencing

Dingani Nkosi[1], Vektra L. Casler[1], Chauncey R. Syposs[1] and Zoltán N. Oltvai[1]

[1] Department of Pathology and Laboratory Medicine, University of Rochester School of Medicine & Dentistry, Rochester, NY 14642, USA

Supplementary Table S1A-B: Gene list for the NGS based assays

Supplemental Table S1A: Oncomine Focus Assay (OFA) hotspot gene list

The Oncomine Focus Assay (OFA) (Thermo Fisher) enables the detection of variants listed in the table of genes, including single nucleotide variants (SNVs) and insertion/deletions (Indels). The limit of detection for SNVs and indels is precise and reproducible at 5% allele frequency with 500X coverage. The limit of detection of indels is less than 25bp. Genes are only interrogated in hotspot regions and are not sequenced in their entirety. Mutations outside the 989 hotspot variants will not be covered by this assay. Variants of uncertain origin (germline versus somatic origin) cannot be determined unequivocally based on sequencing tumor samples alone. The data obtained are analyzed with the Ion Reporter^{TM} software for variant classification and the Torrent Suite Analysis plugin for the coverage analysis. The mutation nomenclature is based on the recommendation from the Catalogue of Somatic Mutations in Cancer (COSMIC, http://cancer.sanger.ac.uk/cosmic).

Gene	Accession #	Exon(s)
AKT1	NM_001014431.1	3
BRAF	NM_004333.4	11,15
DDR2	NM_006182.2	5
ERBB3	NM_001982.3	2,3,6,8,9
FGFR2	NM_000141.4	7-9,12,14
GNAQ	NM_002072.4	4,5
IDH2	NM_002168.2	4
JAK3	NM_000215.3	11,12,15
MAP2K1	NM_002755.3	2,3,6
MTOR	NM_004958.3	30,39,40,43,47,53
PIK3CA	NM_006218.3	2,5,6,8,10,14,19,21
ROS1	NM_002944.2	36,38
AR	NM_000044.3	6,8
CTNNB1	NM_001904.3	3
ERBB2	NM_004448.3	8,17-22
ESR1	NM_001122740.1	9
GNA11	NM_002067.4	4,5
IDH1	NM_005896.2	4
JAK2	NM_004972.3	14
KRAS	NM_033360.3	2-4
MET	NM_001127500.2	14,16,19
PDGFRA	NM_006206.5	12,14,18
RET	NM_020975.4	10,11,13,15,16
ALK	NM_004304.4	21-25
CDK4	NM_000075.3	2
EGFR	NM_005228.4	3,7,12,15,18-21
ERBB4	NM_005235	18

FGFR3	NM_000142.4	7,9,14,16
HRAS	NM_001130442.1	2,3
JAK1	NM_002227.2	14-16
KIT	NM_000222.2	8,9,11,13,17
MAP2K2	NM_030662.3	2
NRAS	NM_002524.4	2-4
RAF1	NM_002880.3	7,12
SMO	NM_005631.4	4,6,8,9

Supplemental Table S1B: TruSeq myeloid panel gene list

The presence or absence of mutations are determined by next generation sequencing (NGS) analysis using the Illumina Truseq Myeloid Panel. This analysis applies only to single base-pair and small insertion/deletion alterations up to 25 base-pairs in the coding regions and adjacent intron/exon boundaries, although it can detect *FLT3* ITDs that are greater than 25-base-pairs. False negative results are also possible due to mutant cell populations below the analytical sensitivity of the method, which is approximately 5% of cells for most mutations, 10% of cells for the common *ASXL1* mutation c.1934dupG and 2% of cells for several hotspot positions including *IDH1* R132, *IDH2* R140 and R172, *JAK2* V617 and exon 12, *MYD88* L265, *NOTCH1* c.7541_7542del, *BRAF* V600E, *KIT* D816, *NRAS* G12, G13 and Q61.

**Gene**	**Accession #**	**Exon(s)**
ASXL1	NM_015338.5	12
CBL	NM_005188.3	8, 9
ETV6	NM_001987.4	all
FLT3	NM_004119.2	14, 15, 20
IDH1	NM_005896.2	4
KIT	NM_000222.2	2, 8-11, 13, 17
MYD88	NM_002468.4	3-5
NRAS	NM_002524.4	2, 3
RUNX1	NM_001754.4	all
SRSF2	NM_001195427.1	1
TP53	NM_000546.5	2-11
ZRSR2	NM_005089.3	all
BCOR	NM_001123385.1	all
CSF3R	NM_156039.3	14-17
EZH2	NM_004456.4	all
GATA1	NM_002049.3	2
IDH2	NM_002168.2	4
KRAS	NM_033360.2	2, 3
NOTCH1	NM_017617.3	26-28, 34

PHF6	NM_032458.2	all
SETBP1	NM_015559.2	4
STAG2	NM_001042749.1	all
U2AF1	NM_001025203.1	2, 6
BRAF	NM_004333.4	15
DNMT3a	NM_022552.4	all
FBXW7	NM_033632.3	9-11
GATA2	NM_032638.4	2-6
JAK2	NM_004972.3	12, 14
MPL	NM_005373.2	10
NPM1	NM_002520.6	12
PTPN11	NM_002834.3	3, 13
SF3B1	NM_012433.2	13-16
TET2	NM_001127208.2	3-11
WT1	NM_024426.4	7, 9

Supplementary Table S2A-D: Mutations detected by Idylla cartridges

Supplemental Table S2A: BRAF cartridge detectable mutations

The Idylla BRAF mutation assay uses an integrated, real-time PCR-based system for qualitative detection of *BRAF* V600 wild type, and V600E, V600E2, V600D, V600K, V600R and V600M mutations.

Protein Change	Nucleotide Change
BRAF V600E	(c.1799T>A)
BRAF V600E2	(c.1799_1800delinsAA)
BRAF V600D	(c.1799_1800delinsAT)
BRAF V600D	(c.1799_1800delinsAC)
BRAF V600K	(c.1798_1799delinsAA)
BRAF V600R	(c.1798_1799delinsAG)
BRAF V600M	(c.1798G>A)
BRAF Wild Type	(c.1799T)

Supplemental Table S2B: EGFR cartridge detectable mutations

The Idylla EGFR mutation assay uses an integrated, real-time PCR-based system for qualitative detection of 52 mutations in exon 18, 19, 20, and 21 of *EGFR*.

GENE	EXON	MUTATION	PROTEIN CHANGE	NUCLEOTIDE CHANGE
EGFR	18	G719A	p.Gly719Ala	c.2156G>C
EGFR	18	G719C	p.Gly719Cys	c.2155G>T
EGFR	18	G719C	p.Gly719Cys	c.2154_2155delinsTT
EGFR	18	G719S	p.Gly719Ser	c.2155G>A
EGFR	19	Deletion 9	p.Leu747_Ala750delinsPro	c.2238_2248delinsGC
EGFR	19	Deletion 9	p.Leu747_Ala750delinsPro	c.2239_2248delinsC
EGFR	19	Deletion 9	p.Leu747_Ala750delinsSer	c.2240_2248del
EGFR	19	Deletion 9	p.Leu747_Glu749del	c.2239_2247del
EGFR	19	Deletion 12	p.Leu747_Thr751delinsPro	c.2239_2251delinsC
EGFR	19	Deletion 12	p.Leu747_Thr751delinsSer	c.2240_2251del
EGFR	19	Deletion 15	p.Glu746_Ala750del	c.2235_2249del
EGFR	19	Deletion 15	p.Glu746_Ala750del	c.2236_2250del
EGFR	19	Deletion 15	p.Leu747_Thr751del	c.2239_2253del
EGFR	19	Deletion 15	p.Leu747_Thr751del	c.2240_2254del
EGFR	19	Deletion 15	p.Leu747_Thr751del	c.2238_2252del
EGFR	19	Deletion 15	p.Glu746_Thr751delinsAla	c.2237_2251del
EGFR	19	Deletion 15	p.Glu746_Thr751delinsIle	c.2235_2252delinsAAT

EGFR	19	Deletion 15	p.Glu746_Thr751delinsVal	c.2237_2252delinsT
EGFR	19	Deletion 15	p.Lys745_Ala750delinsThr	c.2234_2248del
EGFR	19	Deletion 15	p.Glu746_Thr751delinsLeu	c.2236_2253delinsCTA
EGFR	19	Deletion 15	p.Glu746_Thr751delinsVal	c.2237_2253delinsTA
EGFR	19	Deletion 15	p.Glu746_Thr751delinsAla	c.2235_2251delinsAG
EGFR	19	Deletion 15	p.Glu746_Thr751delinsGln	c.2236_2253delinsCAA
EGFR	19	Deletion 15	p.Ile744_Ala750delinsValLys	c.2230_2249delinsGTCAA
EGFR	19	Deletion 18	p.Leu747_Pro753delinsSer	c.2240_2257del
EGFR	19	Deletion 18	p.Glu746_Ser752delinsVal	c.2237_2255delinsT
EGFR	19	Deletion 18	p.Leu747_Ser752del	c.2239_2256del
EGFR	19	Deletion 18	p.Glu746_Thr751del	c.2236_2253del
EGFR	19	Deletion 18	p.Leu747_Pro753delinsGln	c.2239_2258delinsCA
EGFR	19	Deletion 18	p.Glu746_Ser752delinsAla	c.2237_2254del
EGFR	19	Deletion 18	p.Glu746_Ser752delinsAsp	c.2238_2255del
EGFR	19	Deletion 18	p.Glu746_Pro753delinsValSer	c.2237_2257delinsTCT
EGFR	19	Deletion 18	p.Glu746_Ser752delinsIle	c.2236_2255delinsAT
EGFR	19	Deletion 18	p.Glu746_Ser752delinsIle	c.2236_2256delinsATC
EGFR	19	Deletion 18	p.Glu746_Ser752delinsVal	c.2237_2256delinsTT
EGFR	19	Deletion 18	p.Glu746_Ser752delinsVal	c.2237_2256delinsTC
EGFR	19	Deletion 18	p.Glu746_Ser752delinsVal	c.2235_2255delinsGGT
EGFR	19	Deletion 21	p.Leu747_Pro753del	c.2238_2258del
EGFR	19	Deletion 21	p.Glu746_Ser752del	c.2236_2256del
EGFR	19	Deletion 24	p.Ser752_Ile759del	c.2253_2276del
EGFR	20	T790M	p.Thr790Met	c.2369C>T
EGFR	20	S768I	p.Ser768Ile	c.2303G>T
EGFR	20	InsG	p.Asp770_Asn771insGly	c.2310_2311insGGT
EGFR	20	InsASV(9)	p.Val769_Asp770insAlaSerVal	c.2307_2308insGCCAGCGTG
EGFR	20	InsASV(11)	p.Val769_Asp770insAlaSerVal	c.2309_2310delinsCCAGCGTGGAT
EGFR	20	InsSVD	p.Asp770_Asn771insSerValAsp	c.2311_2312insGCGTGGACA
EGFR	20	InsH	p.His773_Val774insHis	c.2319_2320insCAC
EGFR	21	L858R	p.Leu858Arg	c.2573T>G
EGFR	21	L858R	p.Leu858Arg	c.2573_2574delinsGT
EGFR	21	L858R	p.Leu858Arg	c.2573_2574delinsGA
EGFR	21	L861Q	p.Leu861Gln	c.2582T>A

Supplemental Table S2C: KRAS cartridge detectable mutations

The Idylla KRAS mutation assay uses an integrated, real-time PCR-based system for qualitative detection of 21 mutations in exon 2, 3, and 4 of *KRAS*.

Gene	Protein Change	Nucleotide Change
KRAS	G12C	c.34G>T
KRAS	G12R	c.34G>C
KRAS	G12S	c.34G>A
KRAS	G12A	c.35G>C
KRAS	G12D	c.35G>A
KRAS	G12V	c.35G>T
KRAS	G13D	c.38G>A
KRAS	A59E	c.176C>A
KRAS	A59G	c.176C>G
KRAS	A59T	c.175G>A
KRAS	Q61K	c.181C>A; c.180_181delinsAA
KRAS	Q61L	c.182A>T
KRAS	Q61R	c.182A>G
KRAS	Q61H	c.183A>C; c.183A>T
KRAS	K117N	c.351A>C; c.351A>T
KRAS	A146P	c.436G>C
KRAS	A146T	c.436G>A
KRAS	A146V	c.437C>T

Supplemental Table S2D: NRAS-BRAF cartridge detectable mutations

The Idylla NRAS-BRAF-EGFRS492R mutation assay uses an integrated, real-time PCR-based system for qualitative detection of mutations in codons 12, 13, 59, 61, 117, 146 of the *NRAS* gene, in codon 600 of the *BRAF* gene and in codon 492 of the *EGFR* gene (see Table 1: List of detectable variants).

Gene	Protein Change	Variants	Exon
NRAS	G12C	c.34G>T	exon 2
NRAS	G12S*	c.34G>A*	exon 2
NRAS	G12D	c.35G>A	exon 2
NRAS	G12A	c.35G>C	exon 2
NRAS	G12V	c.35G>T	exon 2
NRAS	G13D	c.38G>A	exon 2
NRAS	G13V	c.38G>T	exon 2
NRAS	G13R	c.37G>C	exon 2
NRAS	A59T	c.175G>A	exon 3
NRAS	Q61K	c.181C>A	exon 3
NRAS	Q61L	c.182A>T	exon 3
NRAS	Q61R	c.182A>G	exon 3
NRAS	Q61H	c.183A>C; c.183A>T	exon 3
NRAS	K117N	c.351G>C; c.351G>T	exon 4
NRAS	A146T	c.436G>A	exon 4
NRAS	A146V	c.437C>T	exon 4
BRAF	V600E	c.1799T>A	exon 15
BRAF	V600E	c.1799_1800delinsAA	exon 15
BRAF	V600D	c.1799_1800delinsAC	exon 15
BRAF	V600K	c.1798_1799delinsAA	exon 15
BRAF	V600R	c.1798_1799delinsAG	exon 15
EGFR	S492R	c.1476C>A	exon 12
EGFR	S492R2	c.1474A>C	exon 12

* Due to lower sensitivity, the *NRAS* G12S variant will not be reported.

Supplementary Tables S3-10: Additional mutations in Idylla positive cases

Supplemental Table S3: Cutaneous melanoma specimens with Idylla-identifiable *BRAF* mutations and/or with additional genetic alterations

GENE	Frequency	Idylla positive	Idylla Negative	Cases with extra mutations
BRAF (melanoma)	27	21 (78%)	6 (17%)	8 (30%)
KIT, c.1594G>A, p.Val532Ile, Tier III				
MAP2K1, c.370_371delCCinsTT, p.Pro124Leu,Tier I				
ALK, c.4403G>A, p.Gly1468Glu, Tier III<br>PDGFRA, c.2485G>A, p.Gly829Arg, Tier III				
MAP2K1, c.149_151dupTTG, p.Leu50_Glu51insVal, Tier II<br>JAK3, c.1933C>T, p.H645Y, Tier III				
*BRAF, c.1796_1797insTAC, p.Thr599dup, Tier I				
NRAS, c.35G>A, p.Gly12Asp,Tier I<br>*BRAF, c.1747T>C, p.Phe583Leu, Tier III I				
FGFR2 c.941C>T p.Ala314Val Tier III<br>*BRAF c.1406G>A p.Gly469Glu Tier I				
ROS1 c.5827G>A p.Glu1943Lys Tier II<br>*BRAF c.875C>T p.Ser292Phe Tier III				

***Idylla negative cases**

Supplemental Table S4: Cutaneous melanoma specimens with Idylla-identifiable *NRAS* mutations and/or with additional genetic alterations

GENE	Frequency	Idylla positive	Idylla Negative	Cases with extra mutations
NRAS (melanoma)	28	26 (93%)	2 (7%)	12 (43%)
IDH1, c.394C>T, p.Arg132Cys, Tier I (x3)				
CTNNB1, c.101G>A, p.Gly34Glu, Tier I<br>HRAS, c.38G>A, p.Gly13Asp, Tier I				
MET, c.1076G>A, p.Arg359Gln, Tier III				
FGFR3, c.748C>T, p.Pro250Ser Tier III				
MAP2K2, c.185C>G, p.Ala62Gly, Tier III				
ROS1, c.5917G>A, p.Gly1973Arg, Tier III				
BRAF c.1747T>C p p.Phe583Leu Tier III				
*NRAS, c.181_182delCAinsAG, p.Gln61Arg, Tier I				
*NRAS, c.180_181delACinsTA, p.Gln61Lys, Tier I<br>CTNNB1, c.134C>A, p.Ser45Tyr, Tier I				
GNA11, c.547C>T, p.Arg183Cys, Tier II				

*BRAF,c.1411G>T, p.Val471Phe,Tier I

***Idylla negative cases.**

Supplemental Table S5: Hairy cell leukemia specimens with Idylla-identifiable *BRAF* mutations and/or with additional genetic alterations

**GENE**	**Frequency**	**Idylla positive**	**Cases with additional mutations**
BRAF (hairy cell leukemia [HCL])	11	10 (91%)	3 (27%)
RUNX1, c.155T>A, p.Met52Lys Tier III			
TET2, c.4522delG, p.Ala1508LeufsTer63 , Tier I			
SF3B1, c.1998G>C, p.Lys666Asn, Tier I			

Supplemental Table S6: Colorectal cancer specimens with Idylla-identifiable *BRAF* mutations and/or with additional genetic alterations

**GENE**	**Frequency**	**Idylla positive**	**Idylla Negative**	**Cases with extra mutations**
BRAF (CRC)	53	51 (96%)	2 (4%)	17 (32%)
PIK3CA, c.3140A>G, p.His1047Arg, Tier I<br>AKT1, c.49G>A, p.Glu17Lys, Tier I				
AKT1, c.49G>A, p.Glu17Lys, Tier I<br>ALK, c.3386A>G, p.Glu1129Gly, Tier III				
KRAS, c.35G>A, p.Gly12Asp, Tier I<br>ERBB2, c.3034G>A, p.Asp1012Asn, Tier III				
PIK3CA, c.3140A>T, p.His1047Leu, Tier I<br>PIK3CA, c.3141T>A, p.His1047Gln, Tier II				
PIK3CA, c.1634A>G, p.Glu545Gly, Tier I<br>CTNNB1, c.136C>A, p.Leu46Met, Tier III				
PIK3CA, c.1624G>A, p.Glu542Lys, Tier I				
PIK3CA, c.278G>A, p.Arg93Gln, Tier II				
PIK3CA, c.277C>T, p.Arg93Trp, Tier II<br>PDGFRA, c.2471T>C, p.Val824Ala, Tier III<br>MAP2K1, c.659C>T, p.Ala220Val, Tier III				
PIK3CA, c.1637A>C, p.Gln546Pro, Tier II				

CTNNB1, c.59C>T, p.Ala20Val, Tier III
EGFR, c.874G>A, p.Val292Met, Tier III
PDGFRA, c.2503T>C, p.Cys835Arg, Tier III
KRAS, c.169G>A, p.Asp57Asn, Tier III
PIK3CA, c.2150A>G, p.Asp717Gly, Tier III<br>*BRAF, c.2285C>T, p.Ala762Val, Tier III
NRAS, c.35G>A, p.Gly12Asp, Tier I<br>*BRAF, c.1781A>G, p.Asp594Gly, Tier III
IDH1, c.394C>T, p.Arg132Cys, Tier I<br>PIK3CA, c.1633G>A, p.Glu545Lys, Tier I<br>KRAS, c.35G>T, p.Gly12Val, Tier I<br>*BRAF, c.872_886delTCTCCAAGTTCTTTG, p.Val291_Phe295del, Tier III
PIK3CA, c.278G>A, p.Arg93Gln, Tier II<br>PIK3CA, c.1624G>A, p.Glu542Lys, Tier I<br>ERBB2, c.929C>T, p.Ser310Phe, Tier I

***Idylla negative cases.**

Supplemental Table S7: Colorectal cancer specimens with Idylla-identifiable *KRAS* mutations and/or with additional genetic alterations

GENE	Frequency	Idylla positive	Idylla negative	Cases with extra mutations
KRAS (CRC)	65	62 (95%)	3 (5%)	24 (37%)
PIK3CA, c.112C>T, p.Arg38Cys, Tier I				
PIK3CA, c.3062A>G, p.Tyr1021Cys, Tier I				
CTNNB1, c.134C>T, p.Ser45Phe, Tier I				
PIK3CA, c.3140A>T, p.His1047Leu, Tier I				
PIK3CA, c.1634A>G, p.Glu545Gly, Tier I<br>PIK3CA, c.1624G>A, p.Glu542Lys, Tier I				
PIK3CA, c.1634A>G, p.Glu545Gly, Tier I<br>APC, c.847C>T, p.Arg283Ter, Tier I				
PIK3CA, c.3140A>T, p.His1047Leu, Tier I<br>ERBB2, c.2033G>A, p.Arg678Gln, Tier I				
PIK3CA, c.3140A>T, p.His1047Leu, Tier I<br>CTNNB1, c.121A>G, p.Thr41Ala, Tier I				
IDH1, c.394C>T, p.Arg132Cys, Tier I<br>PIK3CA, c.1633G>A, p.Glu545Lys, Tier I<br>BRAF, c.872_886delTCTCCAAGTTCTTTG, p.Val291_Phe295del, Tier III				
PIK3CA, c.3140A>G, p.His1047Arg, Tier I<br>IDH1, c.314G>A, p.Gly105Asp, Tier III				
ERBB3, c.310G>A, p.Val104Met, Tier I				

FGFR2, c.1196G>A, p.Arg399Gln, Tier III
PIK3CA, c.1132T>C, p.Cys378Arg, Tier II
PIK3CA, c.3139C>T, p.His1047Tyr, Tier II
*KRAS, c.17T>A, p.Leu6His, Tier II
KIT, c.1588G>A, p.Val530Ile, Tier III<br>AKT1, c.138C>A, p.Asp46Glu, Tier III
FGFR3, c.749C>T, p.Pro250Leu, Tier III<br>RET, c.1883C>G, p.Pro628Arg, Tier III<br>AKT1, c.106A>G, p.Ile36Val, Tier III<br>GNA11, c.535G>A, p.Val179Met, Tier III
*KRAS, c.33_34delTGinsAT, p.Gly12Cys, Tier I
BRAF, c.1799T>A, p.Val600Glu, Tier I
ERBB3, c.2329G>T , p.Val777Leu , Tier I
PIK3CA, c.1624G>A, p.Glu542Lys, Tier I
PIK3CA, c.3140A>G, p.His1047Arg, Tier I
BRAF, c.1799T>A, p.Val600Glu, Tier I<br>ERBB3, c.3034G>A , p.Asp1012Asn , Tier III

***Idylla negative cases.**

Supplemental Table S8: Colorectal cancer specimens with Idylla-identifiable *NRAS* mutations and/or with additional genetic alterations

GENE	Frequency	Idylla positive	Cases with additional mutations
NRAS (CRC)	3	2 (66%)	2 (66%)
BRAF, c.1781A>G, p.Asp594Gly, Tier I			
PIK3CA, c.1637A>G, p.Gln546Arg, Tier I<br>PIK3CA, c.3139C>T, p.His1047Tyr, Tier I			

Supplemental Table S9: Lung adenocarcinoma specimens with Idylla-identifiable *EGFR* mutations and/or with additional genetic alterations

Gene	Frequency	Idylla positive	Idylla Negative	Cases with additional mutations

EGFR (Lung adenocarcinoma)	65	56 (86%)	9 (7%)	21(32%)
CTNNB1, c.110C>T, p.Ser37Phe, Tier I				
PIK3CA, c.1624G>A, p.Glu542Lys, Tier I				
BRAF, c.1799T>A, p.Val600Glu, Tier I				
PIK3CA, c.3140A>T, p.His1047Leu, Tier I				
CTNNB1, c.95A>G, p.Asp32Gly, Tier I				
CTNNB1, c.98C>G, p.Ser33Cys, Tier I				
BRAF, c.1803A>C, p.Lys601Asn, Tier I				
EGFR, c.2320G>A, p.Val774Met, Tier II				
EGFR, c.2326C>T, p.Arg776Cys, Tier II				
JAK3, c.1957G>A, p.Val653Met, Tier III				
EGFR, c.2305G>A, p.Val769Met, Tier III				
EGFR, c.847G>T, p.Gly283Cys, Tier III				
KRAS, c.34G>T, p.Gly12Cys, Tier I				
KRAS, c.34G>T, p.Gly12Cys, Tier I<br>*EGFR, c.2386G>A, p.Gly796Ser, Tier II				
PIK3CA, c.1625G>A, p.Glu542Val, Tier I				
KRAS, c.35G>T, p.Gly12Val, Tier I<br>*EGFR, c.1723-1G>C, p.?, Tier III				
FGFR3, c.247C>T, p.Pro83Ser, Tier III<br>EGFR, c.2494C>T, p.Arg832Cys, Tier III				

***Idylla negative cases.**

Supplemental Table S10: Lung adenocarcinoma specimens with Idylla-identifiable *KRAS* mutations and/or with additional genetic alterations

**GENE**	**Frequency**	**Idylla positive**	**Idylla Negative**	**Cases with additional mutations**
KRAS (Lung adenocarcinoma)	158	147(93%)	11 (7%)	25 (16%)
EGFR, c.2156G>C, p.Gly719Ala, Tier I				
BRAF, c.1786G>C, p.Gly596Arg, Tier I				
BRAF, c.1397G>T, p.Gly466Val, Tier I				
PIK3CA, c.3140A>T, p.His1047Leu, Tier I (x4)				
PIK3CA, c.1633G>A, p.Glu545Lys, Tier I (x2)				
CTNNB1, c.110C>T, p.Ser37Phe, Tier I				
PIK3CA, c.3140A>T, p.His1047Leu, Tier I<br>CTNNB1, c.121A>G, p.Thr41Ala, Tier I				

PIK3CA, c.3140A>T, p.His1047Leu, Tier I<br>GNA11, c.547C>T, p.Arg183Cys, Tier I
PIK3CA, c.241G>A, p.Glu81Lys, Tier II
EGFR, c.2386G>A, p.Gly796Ser, Tier II
EGFR, c.1723-1G>C, p.?, Tier III
KIT, c.1960G>T, p.Val654Leu, Tier II
MET, c.3779G>A, p.Gly1260Asp, Tier III
PIK3CA, c.1064A>G, p.Tyr355Cys, Tier III
MAP2K1, c.171G>T, p.Lys57Asn, Tier II
JAK3, c.1631T>C, p.Val544Ala, Tier III
BRAF, c.658G>T, p.Glu220Ter, Tier III
ALK, c.3398G>T, p.Gly1133Val, Tier III<br>IDH1, c.314G>T, p.Gly105Val, Tier III
ALK, c.3572C>A, p.Pro1191His, Tier III<br>ALK, c.3362G>A, p.Gly1121Asp, Tier III
ERBB2, c.2329G>T, p.Val777Leu, Tier I<br>*KRAS, c.37G>T, p.Gly13Cys, Tier I
MAP2K1, c.171G>T, p.Lys57Asn, Tier II<br>*KRAS, c.101C>T, p.Pro34Leu, Tier I

***Idylla negative cases**
